# Reversible and Non-Reversible Transformation of Magnetic Structure in Amorphous Microwires

**DOI:** 10.3390/nano10081450

**Published:** 2020-07-24

**Authors:** Alexander Chizhik, Julian Gonzalez, Arcady Zhukov, Przemyslaw Gawronski, Mihail Ipatov, Paula Corte-León, Juan Mari Blanco, Valentina Zhukova

**Affiliations:** 1 Dept. Phys. Mater., Univ. Basque Country, UPV/EHU, 20018 San Sebastian, Spain; julianmaria.gonzalez@ehu.eus (J.G.); arkadi.joukov@ehu.eus (A.Z.); mihail.ipatov@ehu.eus (M.I.); paula.corte@ehu.eus (P.C.-L.); valentina.zhukova@ehu.eus (V.Z.); 2 Dept. Appl. Phys., Univ. Basque Country EIG, UPV/EHU, 20018 San Sebastian, Spain; juanmaria.blanco@ehu.eus; 3 IKERBASQUE, Basque Foundation for Science, 48011 Bilbao, Spain; 4 Faculty of Physics and Applied Computer Science, AGH Univ. of Science and Technology, 30-059 Krakow, Poland; gawron@newton.fis.agh.edu.pl

**Keywords:** soft magnetic materials, amorphous magnetic wires, magnetic domains, magneto-optic Kerr effect, giant magnetoimpedance effect, magnetic anisotropy

## Abstract

We provide an overview of the tools directed to reversible and irreversible transformations of the magnetic structure of glass-covered microwires. The irreversible tools are the selection of the chemical composition, geometric ratio, and the stress-annealing. For reversible tuning we use the combination of magnetic fields and mechanical stresses. The studies were focused on the giant magnetoimpedance effect and the velocity of the domain walls propagation important for the technological applications. The essential increase of the giant magnetoimpedance effect and the control of the domain wall velocity were achieved as a result of the use of two types of control tools. The performed simulations reflect the real transformation of the helical domain structures experimentally found.

## 1. Introduction

Magnetic wires receive permanent interest because of specific magnetic, electric, and magneto-optical properties, which find the realization in wide gamma of electronic devices [1,2,3,4,5,6].

The importance and novelty of our work is determined basically by the technological application of the microwires. The elucidation of the basic methods for controlling the magnetic structure with the predicted properties is the most promising way for optimizing the operation of magnetic sensors based on wires. We have been dealing with this problem for about 20 years, as evidenced by our first and last work on this topic [5,7].

The nature of glass-coated microwires-circular symmetry, outstanding magnetic properties, and the cheap and accessible fabrication method makes them really attractive from any application’s perspectives. The basic concept of many magnetic sensors is tied up with the well-known phenomena of giant magneto-impedance [8,9,10,11,12]. Also, some of these applications like magnetic logic devices, random access memory, integrated circuits, hard disks, and spintronic [13,14] are fare likely rooted to a fast and controllable domain wall motion or propagation throughout magnetic bistability. So far, glass-coated microwires offer such existence of these two functionalities, either GMI or magnetic bistability, in a broad variety of compositions of each of two main families: Co-based or Fe-based alloys, respectively.

Therefore, control of domain wall (DW) dynamics, amplitude of giant magnetoimpedance effect (GMI), and mechanisms of the magnetization reversal, volume or surface, is the key subject of our investigations. Controlled change of the sign and value of the magnetostriction, the reaching of high DW speeds, and switching from axial Barkhausen jump to circular Barkhausen jump and vice versa are the main roots of the improvement of the magnetic properties related with the technological applications [2,15,16,17].

The approach of our paper is based on the idea to search for methods permitting to control the basic properties of the magnetic microwires. We have focused this paper on these tools influencing on magnetic softness, GMI effect, and single DW velocity in amorphous glass-coated microwires.

Originally, we divided these methods to two groups of reversible and non-reversible changes. These two groups constitute a sequence of actions leading to the expected results. It is evident that the first step of this sequence is the non-reversible change, followed by the finer reversible tuning. Depending on the conditions, different tools could move from one group to another. For example, the torsion stress of the small amplitude causes the reversible changes. The increase of the stress amplitude leads to the un-reversible changes, thereby moving it to the first group. In the same time, the heating causing the annealing process, being lowered in amplitude, leads only to the reversible transformations.

In this work we present the results of the magnetic, magneto-electric, and magneto-optical studies of a series of glass-covered microwires. The studies have been performed in the presence of the different tools induced with reversible and ir-reversible transformation of the magnetic structure. Also, in the frame of the analysis of the experimental results we present the results of the micromagnetic simulations demonstrating the influence of the electric current on the magnetic structure of the microwires.

All of the presented data are our proper experimental results and proper results of the simulations.

## 2. Materials and Methods

To perform the investigation, we have selected six samples from our wide collection of the glass-covered microwires. The selection of each sample was determined by the concrete task related to the studies of reversible and in-reversible transformations of the magnetic structure. In each of the selected samples, these effects manifested themselves most clearly.

There are the samples which were studied:

- №1 Fe_71.7_B_13.4_Si_11_Nb_3_Ni_0.9_, a metallic nucleus diameter of d = 103 μm and a total diameter including the glass coating of D = 158 μm,
- №2 Co_41.7_Fe_36.4_Si_10.1_B_11.8_, diameter of metallic nucleus d = 13.6 μm and total diameter D = 24.6 μm,
- №3 Fe_3.6_Co_69.2_Ni_1_B_12.5_Si_11_Mo_1.5_C_1.2_, d = 22.8 μm, D = 23.2 μm, annealed at T_ann_ = 300 °C for t_ann_ = 1 h,
- №4 Co_67.05_Fe_3.84_Ni_1.44_B_11.33_Mo_1.69_Si_14.47_, d = 21.4 μm, D = 26.2 μm,
- №5 Co_66.87_Fe_3.66_Ni_2.14_Si_11.47_B_13.36_Mo_1.52_C_0.98_ with different diameters of metallic nucleus and total diameters,
- №6 (Co_1-*x*_Mn*_x_*)_75_Si_10_B_15_ (*x =* 0.08, 0.09, and 0.10).

The Taylor-Ulitovsky method (also known as quenching-and-drawing) was used to produce the microwires [18,19].

The production of the glass-coated metal filaments of diameter of about a few microns has been reported by Taylor in 1924 [20]. The method of the fabrication is the following. To produce the microwire the metal is held in a borosilicate glass tube. It is closed at one end of the tube with a diameter of about 10 mm. To soften the glass, the end is heated by a glass flame to a specific temperature. At this temperature, the metal part goes to a liquid state. The glass is drawn down and thin glass capillary containing a metal core produces.

The Taylor and Ulitovsky method was revived by Inoue [21], Chiriac [22], and Larin [23]. The modified production technique was developed, which supplied the composite glass-coated microwires with metallic nucleus of diameter in the range of 1–30 μm and glass covering of 2–10 μm. The key parameters of the modified Taylor and Ulitovsky method were the cooling rate of the metal core, chemical composition, and the stable geometrical ratios along the microwire.

Fabrication process of glass covered microwires causes the fast and simultaneous solidification of a composite structure, which consists of magnetic metallic nucleus and glass quenched from the molten alloy. Essential differences in thermal expansion of the glass and the metal lead to large internal stresses. The magnetic structure of an amorphous glass-coated microwire, in the absence of magnetocrystalline anisotropy, is determined basically by the magnetoelastic coupling energy between the internal stresses and spontaneous magnetization.

The stress induced at the fabrication determines the source of anisotropies. The annealing causes a partial relaxing of the internal stresses induced by the production technique, and gives rise to the induced anisotropy. The controlling of the anisotropy is important for technological applications.

Here we present the influence of the annealing with an example of the microwire №3. We consider the annealing as a tool that causes the ir-reversible transformation. The structure of the as-prepared and annealed microwires was recognized by X-ray diffraction [24,25,26,27].

In our study we have used the following experimental methods:

Flux-metric method was used for hysteresis loops measurements [28]. The magnetic field was produced by a solenoid. The studied sample was placed in a pick-up coil. The normalized magnetization *M/M*_0_ on external field has been obtained. *M* is the magnetic moment in some moment; *M*0 is the magnetic moment at the maximum value of the external field *Hm*. The velocity of DW motion has been measured by Sixtus-Tonks method [29,30].

Originally the Sixtus and Tonks technique consisted of the following elements [31]: a primary coil, which was produced a homogenous-enough magnetic field to induce the propagation of DW wall, short coil induced the nucleation of the DW and two picked up coils. The DW propagation induces the electromotive force in the picked-up coils, which are fixed in the oscilloscope. It was found that in some experimental configurations, the domain wall can nucleate in the middle of the microwire on defects. To avoid mistakes, in the modified set-up we used a system of three pick-up coils [32,33,34,35]. The microwire of 10 cm length is placed coaxially inside of the primary coil. To initiate the DW propagation from the determined end of the wire, one end of the sample was placed outside the magnetization solenoid. The distances between coils were 30 mm.

DW velocity was measured as:

*ν* = *l*/Δ*t* (1)

where time interval Δ*t* is the time difference between the induced peaks and *l* is the space interval between pick-up coils. We can determine the DW velocity sequentially between 3 pick-up coils.

The impedance *Z* was recalculated from the reflection coefficient *S*_11_, which was obtained using network analyzer:

*Z* = *Z*_0_(1 + *S*_11_)/(1 − *S*_11_) (2)

where *Z*_0_ is the impedance of the coaxial line. The GMI ratio Δ*Z/Z* was determined as:

Δ*Z*/*Z* = [*Z*(*H*) − *Z*(*H_max_*)]/*Z*(*H_max_*) (3)

where *H_max_* is the maximum value of the external DC field.

**Figure 1:**
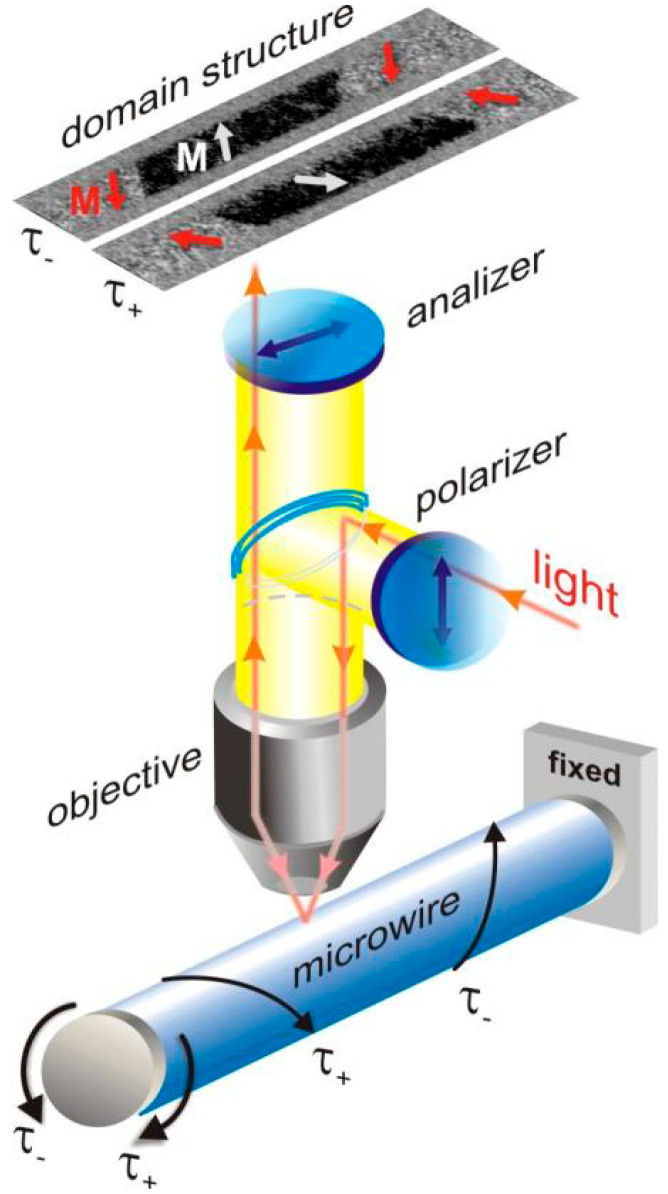
Schematic picture of MOKE geometry and application of torsion stress t_+_ and t_-_. Reprinted with permission from [36].

The images of surface magnetic domain structure were obtained by magneto-optical Kerr effect (MOKE) polarizing microscopy (longitudinal geometry). A combination of crossed axial and circular magnetic fields and external mechanical stresses was applied [36]. The torsion stress τ of two opposite signs and limited range of amplitude was applied to the microwires. One of the wire ends was fixed. The other end was rotary stressed to apply the torsion with different angle α and to change the internal stress distribution (Figure 1). The limits of the stress at which the mechanical failure could appear was not achieved.

## 3. Results and Discussions

### 3.1. Reversible Transformation

Torsion stress and electric current-induced transformations in the sample №1 have been studied using the MOKE technique.

**Figure 2:**
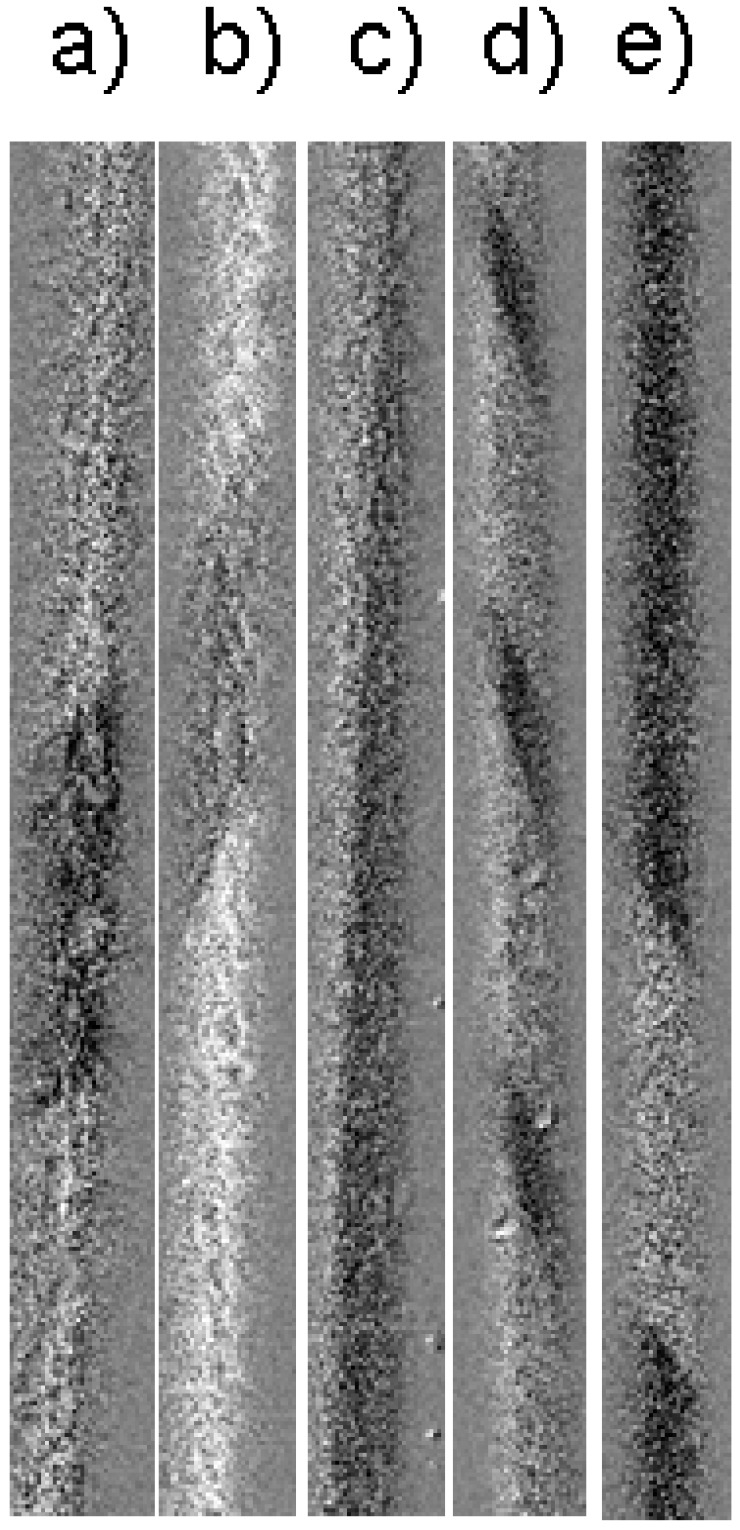
MOKE images of surface magnetic domains. Torsion strain: (**a**) −8 π radm^−^^1^, (**b**) −2 π radm^−^^1^, (**c**) 0, (**d**) 2 π radm^−^^1^, (**e**) 8π radm^−^^1^. Reprinted with permission from [15].

**Figure 3:**
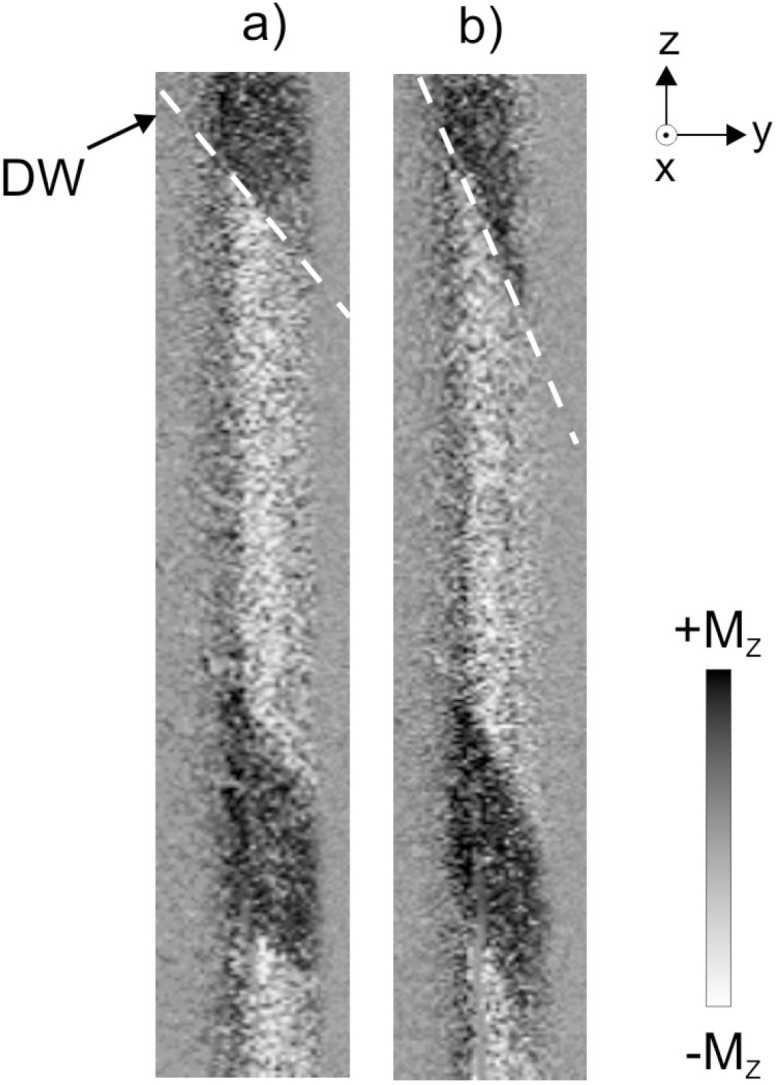
MOKE images of surface magnetic domains. Torsion strain 2πradm^−^^1^. Amplitude of circular magnetic field with amplitude: (**a**) 0, (**b**) 7 A/m. White line shows the inclination of domain walls. Reprinted with permission from [15].

Figure 2 and Figure 3 show the images of magnetic surface domains obtained in the presence of the torsion stress and circular magnetic field. The observed transformations were reversible—when the influence of stress adds circular field was removed, the system returned to its original position.

Figure 2 presents the key details of the effect of the rotation of domain walls induced by torsion stress. There are two types of the helical structure—spiral and elliptical. It was found that the spiral domain structure exists on narrow enough interval of torsion (smaller then 2 πradm^−^^1^). For the higher value of torsion, the elliptical structure exists. The angle of the DW inclination changes with the stress as in the case of spiral and in the case of elliptical structures. The DW with longitudinal orientation exists in the presence of the stress of about 0 πradm^−^^1^ (Figure 2c). Formally, this structure belongs to the spiral type of the structures.

Together with DW rotation the torsion stress causes also the transitions between two types of domain structures: spiral and elliptic. The phase transition is reversible for the torsion smaller than 40 πradm^−^^1^. This value is the limit of the reversibility. In other words, when this value is exceeded during the experiment, the structure does not return to the spiral structure and the phase transition is not observed. 

The circular magnetic field is another tool induced by the reversible transformations and in some experimental situations could be considered as a tool in opposition to the torsion. Figure 3 demonstrates the influence of the circular field. We start in the presence of the torsion stress of 2 πradm^−^^1^ (Figure 2a). Figure 3b shows the structure in the additional presence of a circular field. The rotation of DW is observed in explicit form towards the axial direction, i.e., against the stress. In the same time, the DW could not reach the longitudinal direction because the limit value of the circular field is determined by the limit value of the electric current induced in this field. The value of this limit is determined by the effect of Joule heating. Therefore, the combination “stress—circular field” is considered as a tool of the fine reversible control of helical magnetic structure.

**Figure 4:**
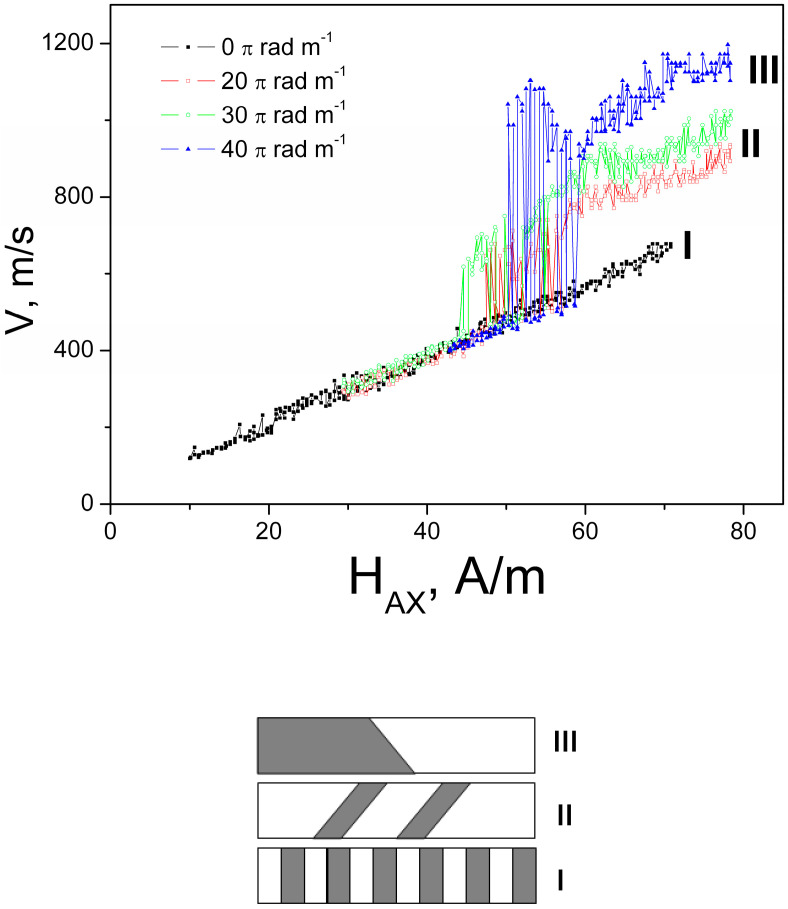
Dependencies of velocity of domain wall on axial magnetic field for different values of torsion stress τ and schematic images demonstrate three types of domain structure. Reprinted with permission from [17].

The dependencies of DM movement on longitudinal magnetic field have been obtained in sample №1 in the presence of small tension stress of about 20 MPa. They were measured by Sixtus–Tonks method. The change of the character of the DW motion as an answer to the torsion application was fixed. The dependencies of the velocity on the longitudinal field in the presence of positive torsion stress are presented in the Figure 4. The similar effect was observed for the negative values of the torsion.

In the absence of the torsion stress, the dependence of the DW velocity is expected and relatively trivial—it is a direct line (black line in the Figure 4). In the presence of the torsion, the noticeable transformation of the velocity dependence takes place. Beginning from the determined value of the stress (about 20 πradm^−^^1^), the clear jump is observed on the velocity dependence. In the presence of relatively small magnetic field, the linear dependence with approximately the same slope were obtained for all values of the stress. The linear character of the velocity dependence is practically maintained after the jump but the value of the jump and correspondingly the velocity depends on the stress (blue, green, and lines). The observed effect was named as “torsion-induced acceleration of domain wall”.

We have focused on the character of the stress induced of the velocity and the reasons of the dependence of the jump on the stress. It should be noted that such a type of the dependences of DW velocity on the field was not observed earlier. In the narrow interval of the external field (around of 55 A/m) the jumping and falling of the velocity value are observed. These jumping and falling velocities were reversible and random.

The additional MOKE study has been performed to clear this effect. Using the MOKE microscopy we tried to find the main types of the surface domain structures having the relation to the observed stress induced acceleration of DW motion. As a result, we have found 3 different types of domain structure, demonstrated schematically in Figure 4.

**Figure 5:**
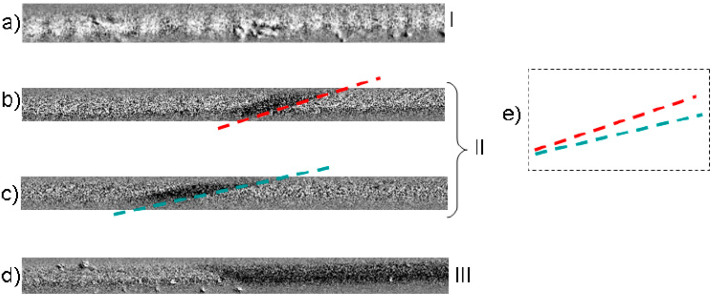
Images of different domain structures obtained in the presence of stress observed for different amplitude of torsion τ: (**a**) 0 πradm^−1^, (**b**) 20 πradm^−1^, (**c**) 30 πradm^−1^, (**d**) 40 πradm^−1^; (**e**) schematically images of spiral structures. Reprinted with permission from [17].

The experimental MOKE images of tree different domain structures obtained in the presence of torsion are presented in Figure 5. Earlier it was demonstrated theoretically the existence of different types of helical structures being in energetically close states. They are characterized by close inclination of the magnetization from the axial axis. Now we understand that the induced jumps in the velocity dependence are in direct relation with the reversible transformation between these structures.

Without torsion stress we observed the domains of type *I*, which have the transversal circular direction of the magnetization. The domain of this type is practically un-sensitive to the torsion. The reason of this effect is the strong correlation these domains with the axially magnetized inner core. There is no contradiction with the structures demonstrated earlier because of small tension stress, which was applied during this experiment.

The spiral domain structure (structure of type *II*) exists in a narrow interval of torsion of 20–30 πradm^−1^. This structure is sensitive on torsion that confirms its independence on the inner core. Spiral structure, along with the elliptic structure, belongs to the helical type of domain structures. We developed some experimental methods, which permit to determine definitely the exact structure of these two observed in the particular experimental conditions.

The increase of the torsion causes elliptic structure (structure of type *II*) forces out the spiral structure from the surface of the microwire. The key difference of these structures is the length of the domain wall. As we know, the domain wall length depends on the angle of the inclination of the domain structure. The elliptic domain wall has a limited length determined by the length of the inclined ellipse. The spiral domain wall has “unlimited” length. Experimentally it is limited only by the length of the studied sample. From the one side, the existence of the elliptic structure is limited by the existence of the stable spiral structure. From the other side, increasing the external stress we reach the natural limit of the angle of the inclination induced by the torsion [37].

The correlation between the domain images presented in Figure 2 and Figure 4 from one side and the velocity dependencies presented in Figure 3 is evident. The stress induced appearance of spiral and elliptical structures recognized in the Figure 3 affects the magnetic field dependence of the DW velocity. Theoretical simulation demonstrates the correlation with the experiment. The induced inclination of the angle of the DW is associated with the DW acceleration.

**Figure 6:**
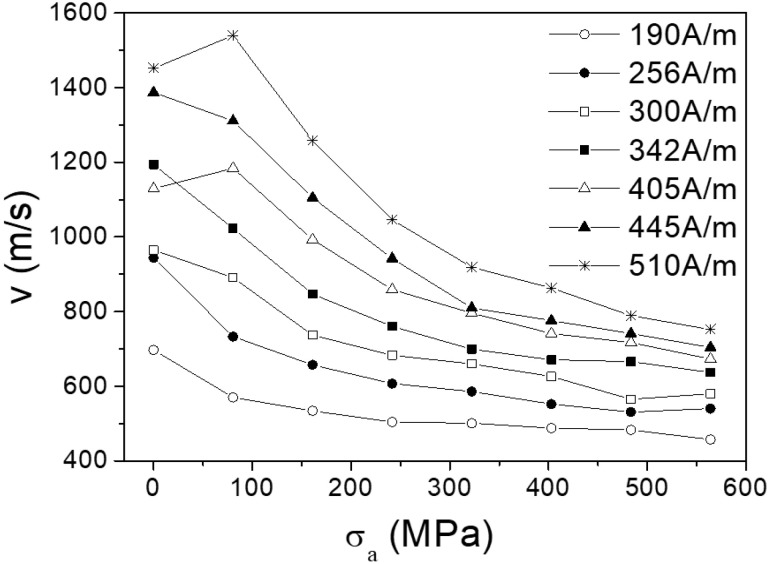
Tension stress dependences of domain wall (DW) velocity in microwires № 2. Reprinted with permission from [6].

The change of the domain wall velocity and mobility under the tension stress was studied in microwires № 2. The dependencies of the velocity on the stress with the magnetic field as a parameter are presented in the Figure 6. DW velocity decreases with the increase of the stress.

**Figure 7:**
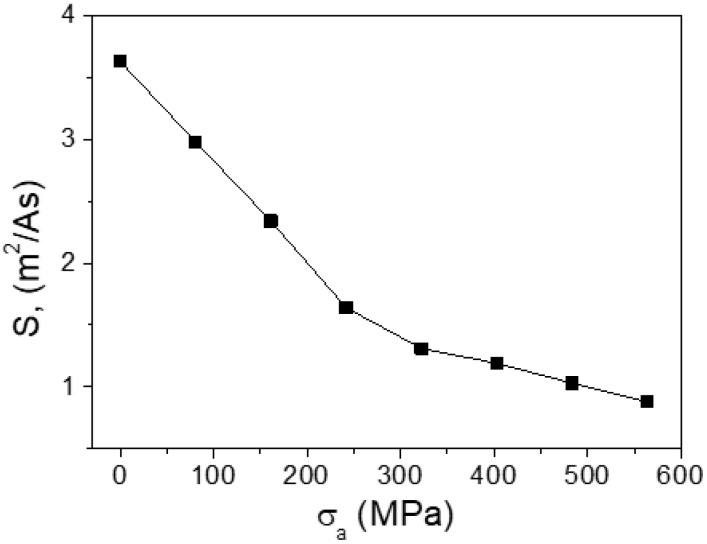
Dependence of DW mobility of microwires № 2 on tension stress. Reprinted with permission from [6].

The DW mobility *S* (Figure 7) was extracted from the dependencies presented in Figure 6. The DW mobility decrease with the increase of the tension stress *σ_a_* observed.

The velocity *v* of the DW movement is determined as:

*v* = *S*(H − H_0_) (4)

where H_0_ is critical propagation field.

For the viscous version of the DW motion the viscosity *S* is determined as:

*S* = 2μ_0_M_s_/β (5)

where β is the coefficient of viscous damping, μ_0_ is magnetic permeability, M_S_ is the saturation value of magnetization. In fact, the key parameter which determines the dynamics of DW is the coefficient of damping.

The important component of the damping β is the magnetic relaxation damping, β_r_. It is associated with the rotation of electron spins. In turn, it is determined by the parameter α Gilbert damping. The parameter α is proportional inversely to the DW wall width δ_w_,

β_r_ = αM_s_/γδ_w_ = M_s_(K_me_/A)^1^^/2^ (6)

where A is the constant of exchange stiffness, K_me_ is the energy of magnetoelastic anisotropy energy, γ is the gyro-magnetic ratio.

This consideration demonstrates the qualitative correlation between domain wall mobility and the magneto-elastic component in the domain wall damping. In other words, when the magnetoelastic anisotropy increases, the domain wall mobility decreases. The achievement of the high mobility of DW requires the decrease of K_me_ constant. To reach it we had to select the composition of the metal nucleus of the microwire with smallest constant of magnetostriction. Another way is the decreasing of internal mechanical stresses by the variation of the geometry of microwire—the relation of the thicknesses of the metallic nucleus and glass covering. The last way is the annealing process causing the relaxation of the internal mechanical stresses.

### 3.2. Ir-Reversible Transformation

#### 3.2.1. Annealing

The annealing treatment allows the reducing of the coercive field and influences on the DW mobility. We consider this effect as ir-reversible. Taking into account the observed single Barkhausen jump during magnetization reversal, it can be assumed that the single domain wall propagation takes place. Consequently, the measurements of the DW velocity using the Sixtus-Tonks method could be realized in stress-annealed Co-rich microwires.

**Figure 8:**
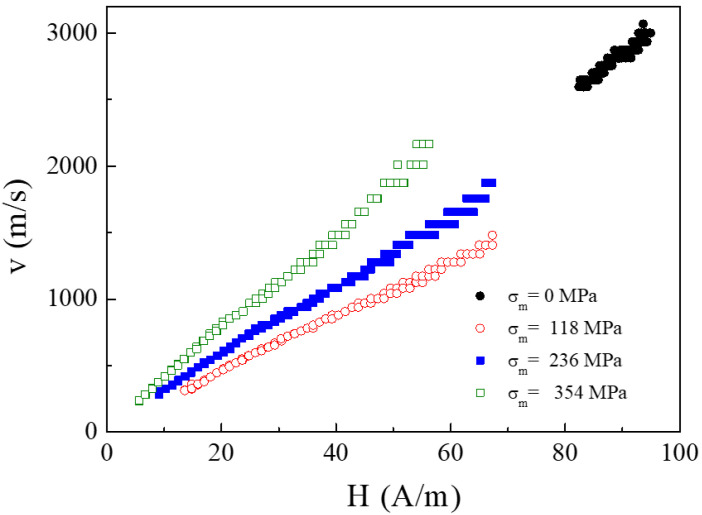
Velocity of DW on axial filed in the presence of different values of the stress applied during annealing. Reprinted with permission from [5].

The series of the microwires № 3 were annealed during 1 h at the temperature of 300 °C in the presence of the tension stress of different values. Figure 8 presents the dependencies of the DW velocity on the external magnetic field for different values of the stress.

The microwires annealed without stress, shows the high enough value of the velocity (about 3 km/s) of DW (see Figure 8). The interval of the external field in which the motion of the single domain wall was fixed short (83–93 A/m).

The DW mobility *S* values are affected by the tension stress *σ_m_*: gradual increase of *S* values (determined as a slope of *v*(*H*) dependencies) is observed upon increase of *σ_m_* (see Figure 8).

**Figure 9:**
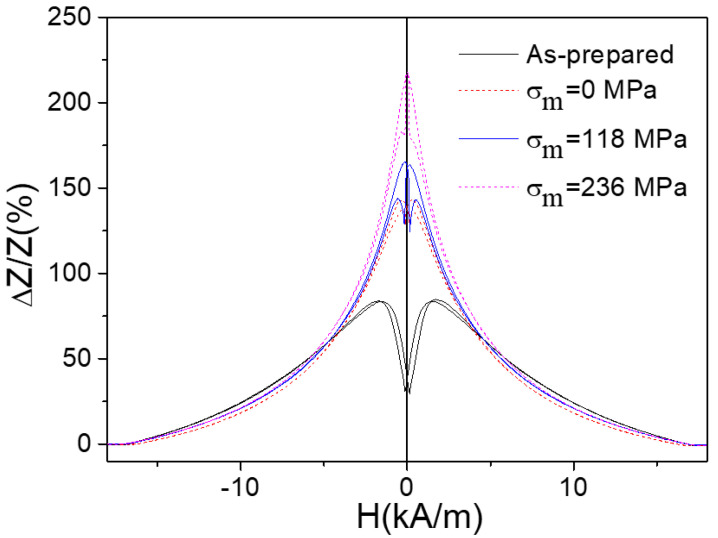
Dependencies of GMI ratio on different value of the external tension stress measured at 200 MHz. Reprinted with permission from [5].

The conditions of the annealing treatment influence also on the GMI effect. Figure 9 presents the transformation of the GMI ration field dependencies caused by the tension stress applied during the annealing process. The increase of the GMI ratio is observed with increase of the value of the stress *σ_m._*

This effect is explained in the frame of the influence of the skin effect on the magneto-electric processes occurring in the magnetic conductor in the presence of the external magnetic field. The bulk hysteresis loops measured by the flux-metric technique provide partial information on the magnetic softness of the whole sample. In the case of the high enough frequency (200 MHz) the GMI ratio characterizes basically the outer layer, which exhibits high circumferential magnetic permeability. In this case, the magnetic softness of the surface layer is more relevant for the GMI effect. The volume of the surface layer is usually much lower than the volume of the inner core and its contribution to the bulk hysteresis loop, generally, is weak.

Here we conclude that the influence of the post-processing on the magnetic processes occurring in the microwires is significant. The variation of the conditions of the annealing is one of the most suitable tools permitted in the predicted optimization of such magnetic properties as domain wall velocity or GMI effect making microwires more suitable for electronic applications.

The field dependencies of the velocity and GMI effect dependencies obtained in Co-rich microwires correlate because the stress applied during the annealing realizes in the stress-induced anisotropy. The stress induced transverse magnetic anisotropy causes the highest GMI effect observed for stress-annealed Co-rich microwires, which present in the same time the rectangular hysteresis loops and hence fast domain wall propagation. The high DW velocity is the result of the fast magnetization switching in the inner axially magnetized single domain.

#### 3.2.2. Geometric Ratio

Magnetic properties of microwires, in particular magnetic anisotropy, are originated basically by the magnetoelastic contribution. In turn, it is determined by the composition of the metal part and by the distribution of the stresses [38,39,40,41]. Because of the glass–metal structure of glass-covered microwires, the internal stresses value is determined by the ratio (metallic nucleus diameter d)/(total diameter D) = ρ. Because of the difference in thermal expansion of metal and glass, the stresses appear during the solidification process, which takes place in the Taylor-Ulitovsky method of microwire production.

**Figure 10:**
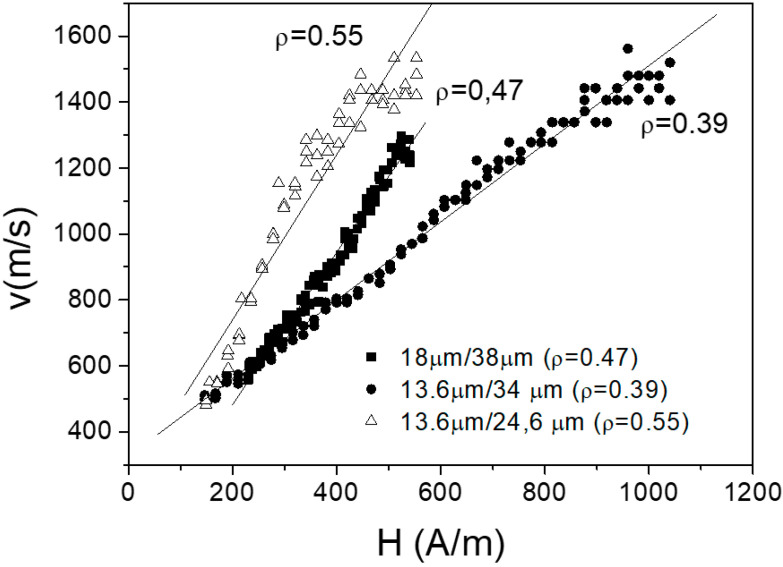
Magnetic field dependences of DW velocity for sample №2 microwires with various ρ ratio. Reprinted with permission from [6].

The external field dependencies of the DW velocity have been measured in the sample №2 for different values of ρ (Figure 10). The different slopes on the velocity dependences are observed for different ρ ratio. As we can see, velocity decreases with the decrease of ρ ratio and in turn with the increase of the internal stresses.

Therefore, the velocity is almost two times higher for the highest ρ ratio: v is about 1500 m/s for ρ = 0.55 and V is about 900 m/s for ρ = 0.39, while the composition is the same. The effect of the magnetoelastic energy has been proved by applying the external stress when we have studied the reversible magnetic transformations.

**Figure 11:**
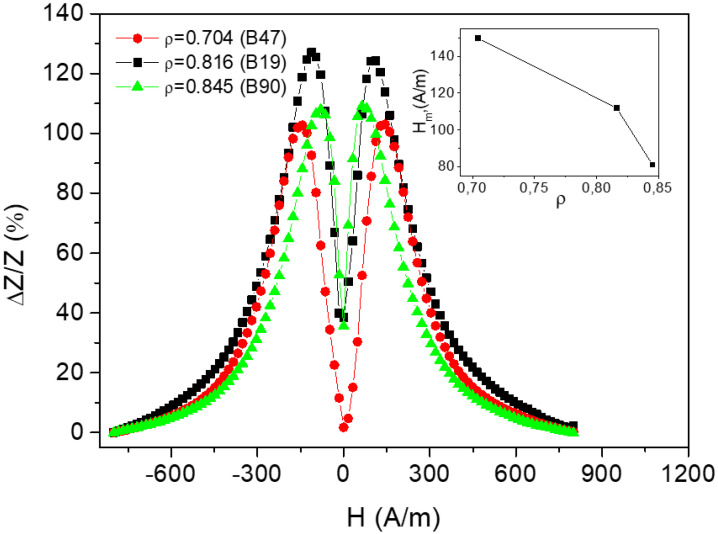
Effect of the sample geometry on magnetic field dependence of giant magnetoimpedance effect (GMI) ratio of microwires №4. Reprinted with permission from [19].

The inset in Figure 11 shows the decrease of the value of the field of GMI maximum with the increasing of the geometric ratio. The influence of the geometric ratio on GMI effect is attributed to the magnetoelastic anisotropy related with the internal stresses. The internal stresses in glass-coated microwires arising from the difference in the thermal expansion coefficients of solidifying metallic nucleus and the covering glass depend in such a way on the ratio between the glass coating thickness and metallic core diameter.

Because the constant of magnetostriction is determined by the chemical composition and has almost zero values in amorphous alloys based on Fe/Co [42,43,44], the observed dependence should be related to the internal stresses.

We could estimate the amplitude of the internal stresses in glass–metal composition because it is caused by the difference in the coefficients of thermal expansion of the glass covering and the metal nucleus. It should be of about 100–1000 MPa. This value is determined equally by the metallic nucleus radius and the thickness of the glass [6].

**Figure 12:**
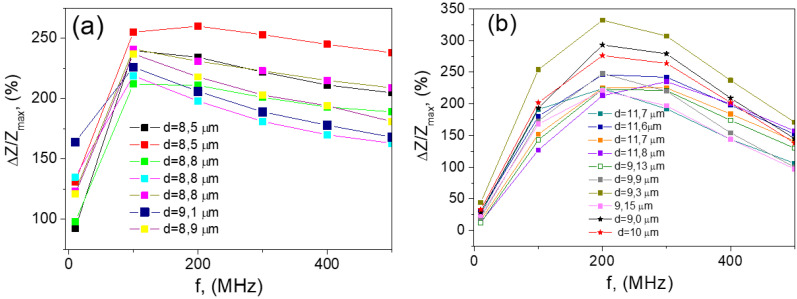
Frequency dependence of microwires №5 with different metallic nucleus diameters. (**a**) metallic nucleus diameters ranging between 8.5 and 9.1 μm; (**b**) metallic nucleus diameters between 9 and 11.7 μm. Reprinted with permission from [19].

Figure 12 demonstrates the GMI effect obtained in a wide range of the frequencies for different diameters of the metallic part of the microwire №5. The highest value of the effect is observed mainly in the range of 100–300 MHz. In the same time, the highest GMI effect takes place and presents at different frequencies for the microwires having the same chemical composition but different geometric parameters. For the wires with d in the range of 9–11.7 μm, the maximum is about 200 MHz, and for the wires with d in the range of 8.5–9 μm the maximum is about 100 MHz.

The change of the position of the maximum H_m_ is related with the effect that H_m_ increases with the frequency. For the high frequency and the high applied magnetic field, H_m_ is approaching to the maximum of the applied field.

At high frequencies, the decreasing of the GMI effect takes place. Therefore, if we have the fixed maximum of external field, the optimum frequency is observed where the highest value of GMI effect takes place. In the same time, the field of GMI maximum is determined by the field of magnetic anisotropy field and we could expect the similar GMI field dependences for similar geometric ratios.

#### 3.2.3. Chemical Composition

The chemical composition also has been selected as a tool permitted for the un-reversible establishment of the magnetic properties in microwires. Sample №6 has been chosen to demonstrate the influence of the chemical composition on the magnetization reversal process. In particular the % part of the Mn has been considered as a tool that has been changed.

**Figure 13:**
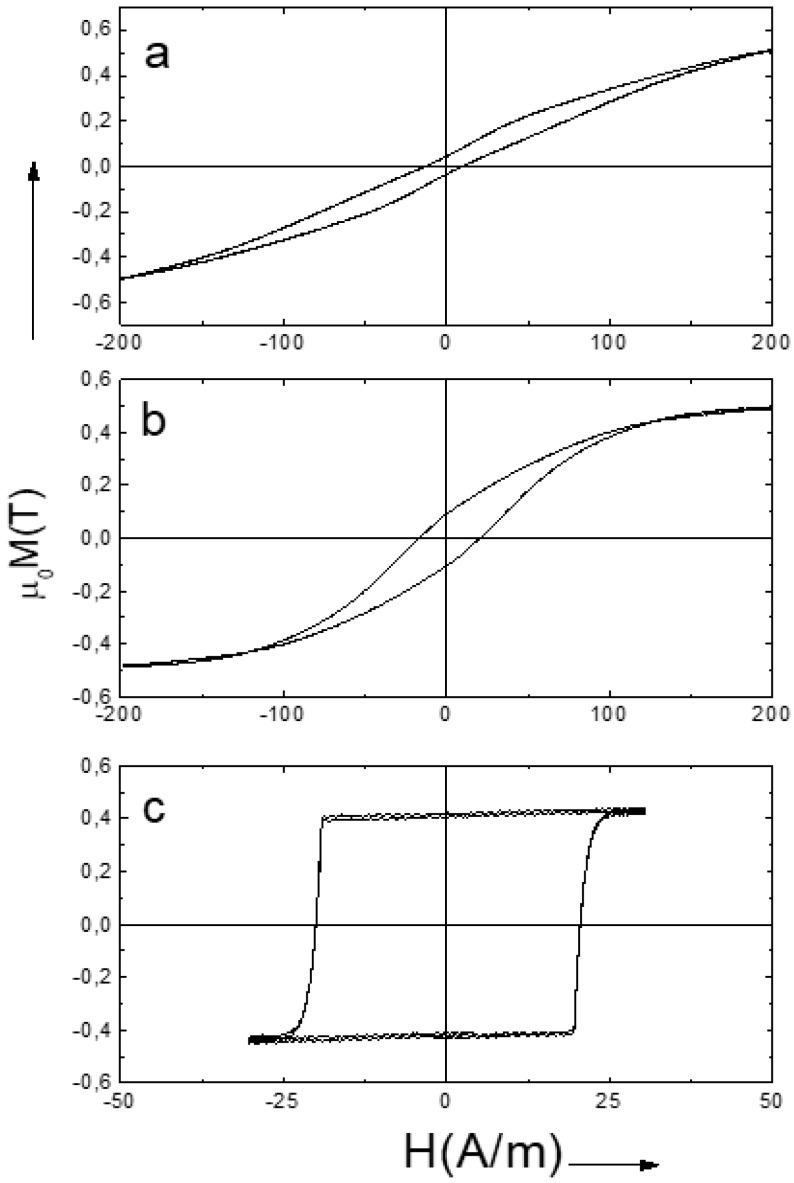
Hysteresis loops the microwire №6. (**a**) x = 0.08, (**b**) x = 0.09, (**c**) x = 0.1. Reprinted with permission from [45].

The magnetic hysteresis obtained for different value of Mn (marked as “x”) are presented in Figure 13. The magnetic behavior shown in the Figure 13a (x = 0.08) confirms the transverse magnetic anisotropy. The coercivity for this case is about 5 A/m. The microwire with x = 0.09 demonstrates the intermediate shape of the hysteresis with coercivity of about 12 A/m. Finally, for x = 0.1 the rectangular shape of the hysteresis loop is observed that confirm the effect of magnetic bistability (coercivity about 24 A/m).

The magnetostriction constant λ_S_ depends on the chemical composition. It changes the sign at Mn concentration value x of 0.09. In spite of low value of λ_S_ (10^−7^), the high enough internal stress causes magnetoelastic anisotropy with transversal easy direction for x = 0.08 (λ_S_ negative) and with longitudinal easy direction for x = 0.1 (λ_S_ positive).

The hysteresis loops take into account the direction of the easy magnetization axis originated by the magnetoelastic anisotropy. For the case of x = 0.08 axial field induced magnetization rotation associated with the transverse direction of the magnetoelastic anisotropy, while for x = 0.1 the hysteresis with rectangular shape reflects the bistability effect possible only for the longitudinal anisotropy.

This consideration is based on the following relation of the magnetoelastic energy and the magnetostrction constant:

K_me_ = 3/2 λ_s_σ_i_, (7)

where σ_i_ are the internal stresses.

To verify this consideration, we have performed the Kerr effect measurements in the same microwire №6 as was studied by the flux-metric technique. The microwire with different concentration of Mn shows the different shape of the transversal and longitudinal MOKE hysteresis loops. The experiments have been performed in circular and axial magnetic fields.

**Figure 14:**
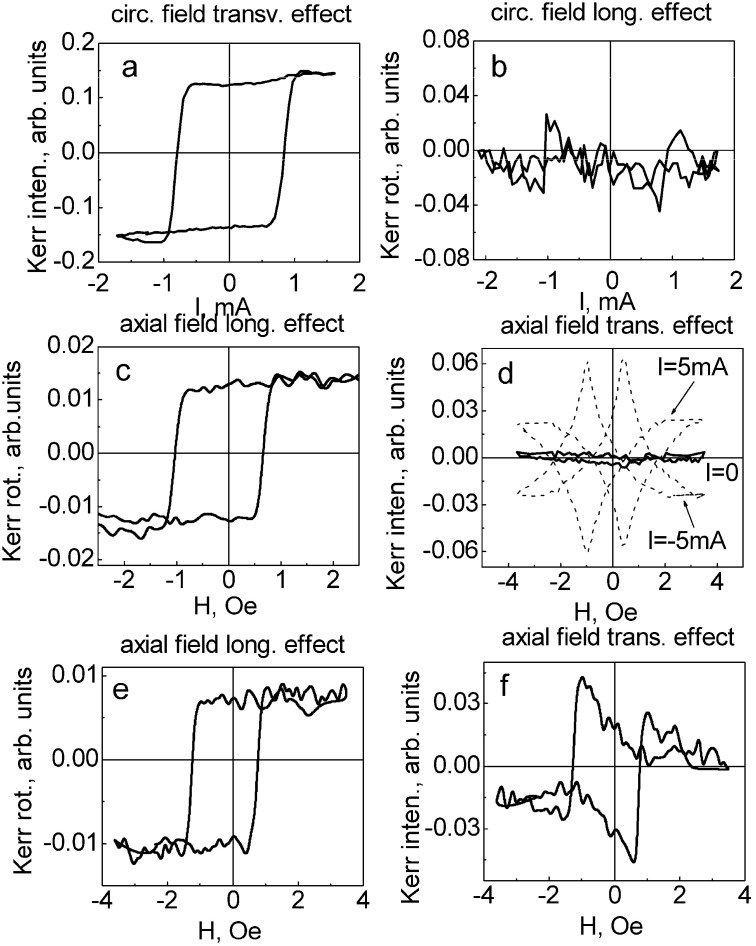
Longitudinal and transverse Kerr effect loops of microwire №6 for different Mn content: (**a**,**b**) x = 0.07; (**c**,**d**) x = 0.11; (**e**,**f**) x = 0.09. Reprinted with permission from [37].

For the case of x = 0.07, the MOKE hysteresis has been obtained in the circular magnetic field (Figure 14a,b). The rectangular shape of hysteresis in Figure 14a means the jump in circular direction. The peaks in longitudinal direction at the same fields confirm the large Barkhausen jump related to the effect of circular bistability. This hysteresis is in agreement with the flux-metric hysteresis presented in Figure 13a.

At the same time, for x = 0.11 the rectangular MOKE hysteresis is observed as an axial magnetic field (longitudinal effect, Figure 14c). This shape reflects the axial large Barkhausen jump. Figure 14d (transversal effect) demonstrates a more complicated experiment: the DC circular field of two opposite directions has been added. Without circular field there is no MOKE signal. This effect is related to the magnetization reversal realized as the motion of domain walls between the axial domains. In the presence of the circular field the signal appears because of the field induced inclination of the magnetization from the axial direction.

The microwire with x = 0.09 (Figure 14e,f) is similar to the case of x = 0.11, but some difference should be noted. While the longitudinal MOKE hysteresis in axial field is rectangular, the transversal loop is associated with sharp nucleation of domains and rotation of the magnetization. All of this could be attributed to the effect of helical bistability.

As was mentioned above, the magnetic structure and the magnetization reversal could be associated with sign and the value of the λ_s_ constant. It was independently determined [46] that λ_s_ is negative for x < 0.1 and is positive for x > 0.1. The MOKE experiments are in agreement with this independent study taking into account that longitudinal magnetization in the outer shell for x = 0.11 is related to positive λ_s_ and for x = 0.07 the circular magnetization is related to the negative λ_s_.

## 4. Simulation

For the first time, the calculations of the helical structure in microwires were presented in [47] and were developed in [48]. Here we present the new combination of the hysteresis and images.

There is a procedure of the simulations. The directions and the values of the magnetization were calculated in discrete points of domain structure. Blue and red arrows show the direction of magnetization in the spiral domains.

The blue arrows with maximum intensity correspond to the left direction. This means that the magnetization component is directed along the microwire axis. The red arrows correspond to the right direction. This means that magnetization component is directed along the microwire axis but in the opposite direction then in the plus one case. If the axial magnetization is zero, the color is white and the arrow is perpendicular to the axis of the microwire. Different intensity of the colors means different values of the axial component of the magnetization or different angles of inclination of the arrows in relation to the axis of the microwire.

**Figure 15:**
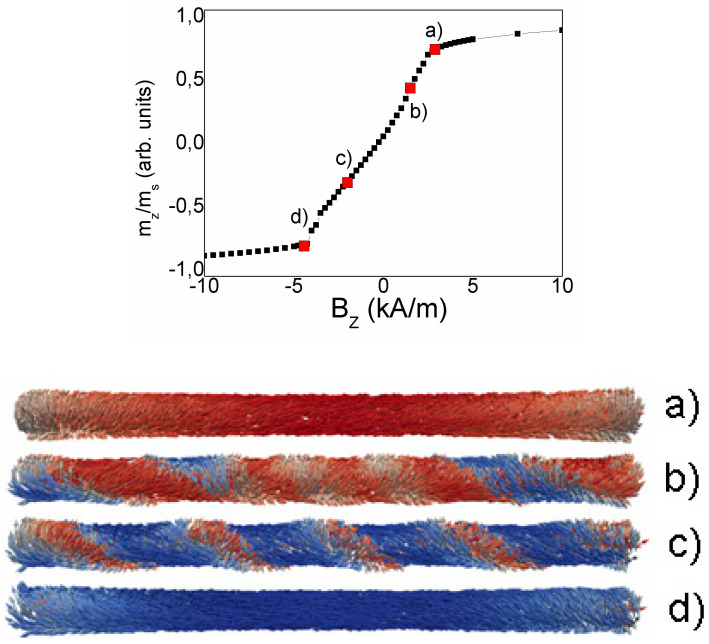
Calculated magnetization reversal curve and the images of spiral domains in cylinder with 1 μm diameter and 15 μm length. Images (**a**–**d**) correspond to the points (**a**–**d**) in the hysteresis. Electric current is 1 mA.

**Figure 16:**
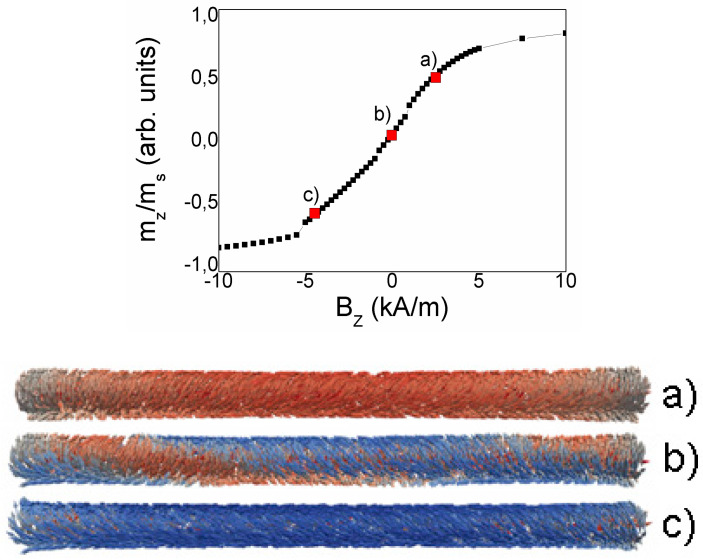
Calculated magnetization reversal curve and the images of spiral domains in cylinder with 1 μm diameter and 15 μm length. Images (**a**–**c**) correspond to the points (**a**–**c**) in the hysteresis. Electric current is 7 mA.

Figure 15 and Figure 16 present the results of the micromagnetic simulations of the magnetization reversal curves and surface domain images in the microwire. To demonstrate the role of the circular magnetic field, the calculation was performed in the presence of electric current flowing along the microwire to produce the circular field. The amplitude of current does not exceed the critical value related to the overheating and the current is considered as a parameter which induces the reversible changes of the magnetic structure.

The calculations were performed in a magnetic cylinder with discretization of 10 × 10 × 5 nm. In the simulations performed with the help of the mumax program [49], the constant A was taken as 20 pJ/m and the saturation magnetization μ_0_M_s_ was equal 0.7T. The magnetoelastic anisotropy is approximated as uni-axial with its spatial dependence related to the experimental stress distribution in a microwire [50]. The magnetoelastic constant was determined using the mentioned above formula (7). The value of magnetostriction constant was 2 × 10^−7^.

The simulated magnetic cylinder is subjected to two magnetic fields: variable axial field and circular Oersted field. The external field was changed gradually along the cylinder axis. The initial state was the saturation marked as m_z_/m_s_ = 1 and the finish state was the saturation marked as m_z_/m_s_ = −1. The control parameter of the simulation was the circular field, which has a distribution in accordance with the Amper’s formula.

Figure 15 and Figure 16 present the magnetization reversal curves and the images of the surface domains. The calculations were performed for a current of 1 mA (Figure 15) and 7 mA (Figure 16).

The magnetization process begins with the formation of vortices at the ends of the microwire. The vortices, as shown in Figure 8, have the opposite chirality. A decrease in the value of the external applied field causes an inclination of magnetic vectors in the entire volume of the microwire and forms spiral domains. The spiral structure gradually increases from both ends of the microwire towards the center of the microwire (see Figure 15b,c) according to the chirality defined by the vortices. The spiral domain structure is not a formation that occurs only on the surface of the microwire, but also occupies part of the volume inside the microwire. When the spiral is fully formed and extends to the length of the entire microwire, further reducing the value of the applied field, leads to a phenomenon that can be termed spiral propagation. Areas where the magnetic moments are set in accordance with the applied field increase, causing the spiral to move and at the same time unscrew. Eventually, the spiral structure disappears from the surface (see Figure 15d) and its remains in the form of a tube penetrate inside the microwire. Further reduction of the field value also results in setting the magnetic moments inside the microwire according to the field.

At zero circular field, the spiral domain structure consists of two parts with: left- or right-hand chirality. Under applied circular magnetic field, the chirality of the spiral structure becomes uniform in the whole wire, with the direction depended on the direction of the current. If we compare the figures for current of 1 and 7 mA, we will see that an increase of the circular field inclines the magnetic vectors towards the circular direction, perpendicular to the axis of the microwire. It causes a reducing of the density of the spiral structure, (see Figure 15b and Figure 16b), as well as a change of its inclination angle.

The problem of the influence of DW structure on the movable properties of DW has been analyzed in detail in our article [48]. Here, the spiral domain wall demonstrates experimentally the increase of the DW mobility with the increase of the angle of the DW inclination. In turn, the present calculations demonstrate the correlation between the angle of the induced inclination and length of the DW. This result of the simulation could be considered as the most essential result.

## 5. Conclusions

Here we present the tools that could produce the reversible and irreversible transformations in the glass-covered magnetic microwires. Irreversible transformations are the first step transformations that permit to select and fix the main predicted magnetic and electric properties. From the one side, these properties could not be changed posteriori, but form the other side, they could serve the basis of the stable and repeatable operation of the technological technique. At the second stage we apply the tools caused by the reversible transformations that serve for the fine tuning. This tuning is realized when the conditions of the technique functioning changes.

One of the main tools causing the irreversible transformation is annealing in the presence of stress. The annealing with stress is effective for Co-rich glass-covered microwires. We have observed more than doubled enhancement of GMI ratio. Also, we observed an increase of the DW velocity of about one and a half times in Co-rich microwires. Therefore, the annealing with stress Co-rich microwires demonstrate simultaneously fast domain wall motion and high GMI effect.

The selection of the chemical composition and changing of the thickness of glass covering of the microwire are also considered as tools caused by the irreversible transformation of the magnetic structure that was realized in around two times variation of the DW mobility. Controlling the sign of the magnetostriction, we could obtain the microwires with the longitudinal or transversal large Barkhausen jump.

For reversible tuning, we use the circularly directed external magnetic field and external stresses-tension and torsion. The change of the sign and the amplitude of the torsion induced the change of the direction and the amplitude of the DW inclination. The torsion stress could induce the reversible transition between two types of helical structures: spiral and elliptic which have almost two times different DW mobility in Fe-rich microwires.

In the relation of the tension stress, to get the high domain wall velocity and mobility, the magnetoelastic energy should decrease. This can be achieved by the application of the external stresses and by the control of the distribution of the internal stresses.

The simulations confirm the observed experimental rotation of the helical domain walls induced by the electric current, demonstrating in such a way the correlation between the MOKE experiments and the calculations.
